# Varietal Differences in Juice, Pomace and Root Biochemical Characteristics of Four Rhubarb (*Rheum rhabarbarum* L.) Cultivars

**DOI:** 10.3390/biotech12010012

**Published:** 2023-01-19

**Authors:** Viktor Kharchenko, Nadezhda Golubkina, Alessio Tallarita, Maria Bogachuk, Helene Kekina, Anastasia Moldovan, Vladimir Tereshonok, Marina Antoshkina, Olga Kosheleva, Sergey Nadezhkin, Gianluca Caruso

**Affiliations:** 1 Federal Scientific Vegetable Center, 143072 Moscow, Russia; 2 Department of Agricultural Sciences, University of Naples Federico II, 80055 Naples, Italy; 3 Federal Center of Nutrition and Biotechnology, 109240 Moscow, Russia; 4 Department of Hygiene, Medical Postgraduate Academy, 123995 Moscow, Russia; 5 Educational-Experimental Soil-Ecological Center, Lomonosov Moscow State University, 119991 Moscow, Russia

**Keywords:** rhubarb, juice, pomace, root, antioxidants, dietary fiber, pectin

## Abstract

The complex evaluation of varietal biochemical differences in rhubarb juice, pomace and roots is highly useful to develop an efficient processing technology. Research was carried out to compare four rhubarb cultivars (Malakhit, Krupnochereshkovy, Upryamets and Zaryanka) in terms of the quality and antioxidant parameters of juice, pomace and roots. The laboratory analyses showed a high juice yield (75–82%) with a relatively high content of ascorbic acid (125–164 mg L^−1^) and other organic acids (16–21 g L^−1^). Citric, oxalic and succinic acids accounted for 98% of the total acids amount. The juice of the cultivar Upryamets demonstrated high levels of the natural preservatives sorbic (36.2 mg L^−1^) and benzoic acids (11.7 mg L^−1^), which are highly valuable in juice production. The juice pomace proved to be an excellent source of pectin and dietary fiber, whose concentrations reached 21–24% and 59–64%, respectively. The total antioxidant activity decreased according to the following sequence: root pulp (161–232 mg GAE g^−1^ d.w.) > root peel (115–170 mg GAE g^−1^ d.w.) > juice pomace (28.3–34.4 mg GAE g^−1^ d.w.) > juice (4.4–7.6 mg GAE g^−1^ f.w.), suggesting that root pulp is a highly valuable antioxidant source. The results of this research highlight the interesting prospects of the complex rhubarb plant processing for the production of juice, containing a wide spectrum of organic acids and natural stabilizers (sorbic and benzoic acids), dietary fiber and pectin (juice pomace) and natural antioxidants (roots).

## 1. Introduction

Lately, several investigations have been devoted to garden rhubarb (*Rheum rhabarbarum* L.) both as a promising source of biologically active compounds and an agricultural crop, which is highly valuable in the food industry [1]. Indeed, this perennial plant is highly popular for production of juice, jams and confectionery and alcoholic beverages, including bitter liqueur with rhubarb rhizomes [1]. This species belongs to the Polygonaceae family which combines about 60 rhubarb species, many of which are highly valuable and are still officially included in the Chinese, Korean and Japanese Pharmacopoeia [2]. Rhubarb may be considered as a fine example of Hippocrates’s words: “Let food be thy medicine and let the medicine be food”. Indeed, these plants have been known for centuries as medicinal plants for their health-promoting effects, and their stems are known for their antitumor [3], anti-inflammatory [4,5], wound-healing and fever-relieving properties, osteoporosis prevention, regulation of gastrointestinal flora [6] and heart protection [7]. Rhubarb roots demonstrate high antioxidant activity [8], showing beneficial effects against diabetes, hypertension, obesity [9] and ulcers [10].

Garden rhubarb (*R. rhabarbarum*) is common in Europe, North America and a part of Asia [2] and may be grown both in greenhouse and in the field. Rhubarb stalk biomass may be regulated via bud dormancy during winter, while spring growth is characterized by intensive changes in hormonal status, sugar and polyphenols accumulation [11,12]. A comparison of different agrochemical growth approaches for *R*. *rhabarbarum* growth and development revealed that chemical fertilization is highly effective to improve garden rhubarb yield, while organic management provides the highest AO profile of plants [13].

The mosaic investigations carried out on garden rhubarb biological activity have indicated its antiviral and antimicrobial [14,15], anticancer [16,17], antioxidant [18], anti-inflammatory [19,20] and cardioprotective [19,21] properties. Garden rhubarb is highly valued in traditional medicine in Hungary [22], Germany [23] and Korea [24].

The biological activity of *Rheum rhabarbarum* is connected with high levels and the joint effect of anthraquinones, polyphenols, stilbenes and organic acids [25]. Investigations of *R. rhabarbarum* biochemicals revealed volatile compounds in stems [26], various polyphenols in methanol extracts of rhizomes [27] and stalks [28], ethylacetate rhizome extracts [29] and fresh juice [1]. Anthraquinones were identified in rhizomes water [30], methanol extracts [31] and leaves [29]. Stylbens were separated from rhizome methanol extracts [31].

The importance of garden rhubarb for human health and nutrition has promoted various investigations addressed to optimize juice production [1,32], root antioxidant characteristics [28], pharmaceutical potential [25] and utilization in the food industry [1]. On the other hand, the complex characterization and processing of rhubarb plants have not been performed so far, also due to high levels of oxalic acid, which is toxic for human organisms at remarkable concentrations because of calcium oxalate precipitation. In this respect, rhubarb leaves are especially dangerous and should not be used for nutritional purposes [33]. The mosaic data regarding species [25,34,35] and varietal differences [28,33,36] in the accumulation of biologically active compounds in rhubarb hamper the development of efficient processing technology. Notably, up to date rhubarb plant parts have been analyzed separately, paying special attention to the biochemical and medicinal properties [2,37] of roots [38,39], stalks [11,12,36] or juice [1]. Indeed, the adequate comparison between different rhubarb cultivars and species relevant to the prospects of their complex utilization is still unavailable.

The present investigation aimed to evaluate chances to manage the complex rhubarb processing and to identify varietal differences in juice, pomace and root quality parameters.

## 2. Materials and Methods

### 2.1. Experimental Protocol and Growing Conditions

The research was carried out at the experimental fields of the Federal Scientific Vegetable Center, Moscow region (55°39.51′ N, 37°12.23′ E), in the years 2021 and 2022 to compare four rhubarb cultivars, Malakhit, Krupnochereshkovy, Upryamets and Zaryanka, that were selected at the mentioned Center. A randomized complete block design was used with three replicates. Six-year-old plants were grown in sod–podzolic clay–loam soil, pH 6.8, 2.1% organic matter, 1.1 g·kg^−1^ N, 0.045 g·kg^−1^ P_2_O_5_, 0.357 g·kg^−1^ K_2_O, spaced 80 × 70 cm. The plants were harvested at the beginning of June, after which samples were taken from all the plots and transferred to the laboratory, where the leaves, stalks and roots were separated from each other. The roots were washed with water to remove the soil particles and were dried with filter paper, and the peel and pulp were separated. The stalks were homogenized and used for juice production using Robot coupe J 80 Ultra (Robot Coupe. Vincennes Cedex—France). The pomace and roots were dried at 70 °C to a constant weight, and then they were homogenized.

### 2.2. Juice Density and Brix

The juice density and Brix° were determined using a 25 mL pycnometer and Abee Refractometer, respectively, as described by Ramasami et al. [40].

### 2.3. Dry Matter

The dry matter was assessed gravimetrically by drying the samples in an oven at 70 °C until they reached a constant weight.

### 2.4. Anthocyanins

The anthocyanin content was determined via differential spectrophotometry by using a Unico spectrophotometer (Unico 2804 UV, Suite E, Dayton, NJ, USA) with the absorption values of the methanolic extracts at 520 nm at pH 3.5 and 1.0 [41]. The anthocyanin (Ac) concentration (in mg-eq cyanidine-3-glucoside L^−1^) was calculated according to the formula

Ac = (ΔD × 449 × V × 1000):(26,900 × a),(1)

where ΔD is the difference in light absorption (520 nm) between the extract at pH 1.0 and pH 3.5, 449 is the molecular mass of cyanidine-3-glucoside, V is the extract volume in mL, 1000 is the conversion factor to 1000 mL of juice, 26,900 is the cyanidine-3-glucoside extinction value and ‘a’ is the sample weight in g.

### 2.5. Preparation of Ethanolic Extracts

A total of 2 mL of rhubarb juice, or one gram of dry root/pomace powder were extracted with 20 mL of 70% ethanol at 80 °C for 1 h. The mixture was cooled and quantitatively transferred to a volumetric flask, and the volume was adjusted to 25 mL. The mixture was filtered through filter paper and was further used to determine the polyphenols and total antioxidant activity.

### 2.6. Polyphenols (TP)

The total polyphenols in the rhubarb roots, juice and pomace were determined in 70% ethanol extracts using the Folin–Ciocalteu colorimetric method as previously described [42]. A total of 1 mL of ethanolic extract, prepared according to Section 2.5, was transferred to a 25 mL volumetric flask, to which 2.5 mL of saturated Na_2_CO_3_ solution and 0.25 mL of diluted (1:1) Folin–Ciocalteu reagent were added. The volume was brought to 25 mL with distilled water. One hour later, the solutions were analyzed with a spectrophotometer (Unico 2804 UV, Suite E, Dayton, NJ, USA), and the concentration of polyphenols was calculated according to the absorption of the reaction mixture at 730 nm. As an external standard, 0.02% gallic acid was used. The results were expressed as mg of Gallic Acid Equivalent per g of dry weight (mg GAE g^−1^ d.w).

### 2.7. Antioxidant Activity (AOA)

The antioxidant activity of roots, juice and pomace was assessed using a redox titration method according to Golubkina et al. [42] via the titration of the 0.01 N KMnO_4_ solution with ethanolic extracts of the samples, produced as described in Section 2.5. The reduction of KMnO_4_ to colorless Mn^+2^ in this process reflects the quantity of antioxidants that were dissolvable in 70% ethanol. The values are expressed as mg Gallic Acid Equivalents (mg GAE g^−1^ d.w.).

### 2.8. Total Dissolved Solids (TDS)

The TDS was determined in water extracts using a TDS-3 portable conductometer (HM Digital, Inc., Seoul, Korea).

### 2.9. Nitrates

Nitrates were assessed with an ionomer Expert-001 (Econix Inc., Moscow, Russia) equipped with an ion-selective electrode according to Kharchenko et al. [43]. A total of 5 g/mL of fresh rhubarb juice, pomace and roots were homogenized with 50 mL of distilled water. A total of 45 mL of the resulting extract were mixed with 5 mL of the 0.5 M potassium sulfate background solution (needed to regulate the ionic strength) and analyzed with an ionomer for nitrate determination.

### 2.10. Organic Acids

The organic acids in rhubarb juice were separated using the capillary electrophoresis of juice water extracts by Kapel-105M (Lumex, St. Petersburg, Russia) and determined via the light absorption of components at 254 nm according to [44]. Appropriate standards of organic acids (citric, oxalic, succinic, ascorbic, acetic, lactic, malic, tartaric, sorbinic, benzoic) were obtained from Sigma Aldrich (Burlington, MA, USA). The organic acid concentrations (X) in g L^−1^ were assessed according to the following formula:

X = k × C(2)

where k is the dilution coefficient and C is the organic acid concentration, obtained from a calibration curve in g L^−1^.

The results were expressed as the mean of three replications.

### 2.11. Dietary Fiber

The dietary fiber content in the rhubarb juice and pomace was assessed gravimetrically after the enzymatic sequential hydrolysis of starch and non-starch compounds with α-amylase, protease and amyloglucosidase (Sigma Chemical Co., St. Louis, MI, USA) to mono-, di-, oligosaccharides and peptides and the appropriate precipitation of dietary fibers with ethanol. The fiber content was determined after drying the precipitate at 70 °C to a constant weight and was expressed in % per d.w. [45].

### 2.12. Pectin

The content of pectin in the rhubarb pomace was evaluated gravimetrically after extraction with 0.05 M hydrochloric acid in a water bath and ethanol precipitation [46]. A total of 1 g of dry homogenized rhubarb pomace was mixed with 0.05 M HCl and heated at 95 °C for 30 min. After cooling, the precipitate was separated and the extraction was repeated. The combined water extracts were mixed with 1.5 volumes of ethanol, acidified with hydrochloric acid (2 mL per 1 L of ethanol) and left at room temperature for pectin precipitation. Half an hour later, the pectin was separated via filtration and was washed with water to remove traces of hydrochloric acid. The resulting residue was dried at 70 °C to a constant weight. The pectin content was assessed as the mean of three replications and was expressed in % per pomace dry weight.

### 2.13. Statistical Analysis

Data were processed by analysis of variance, and mean separations were performed through the Duncan’s multiple range test, with reference to a 0.05 probability level, using SPSS software version 21. The data expressed as percentages were subjected to an angular transformation before processing.

## 3. Results and Discussion

### 3.1. Yield and Morphological Characteristics

Rhubarb plants are priority appreciated for stalks, which have a high nutritional value and biological activity [2,25]. The data presented in Table 1 indicate that among the rhubarb cultivars tested, cv. Upryamets had the highest stalk yield, which exceeded that of the other cultivars by 1.4–1.8 times (Table 1) and was close to the values of five Slovak cultivars reported by Mezeyova et al. [32].

**Figure 1:**
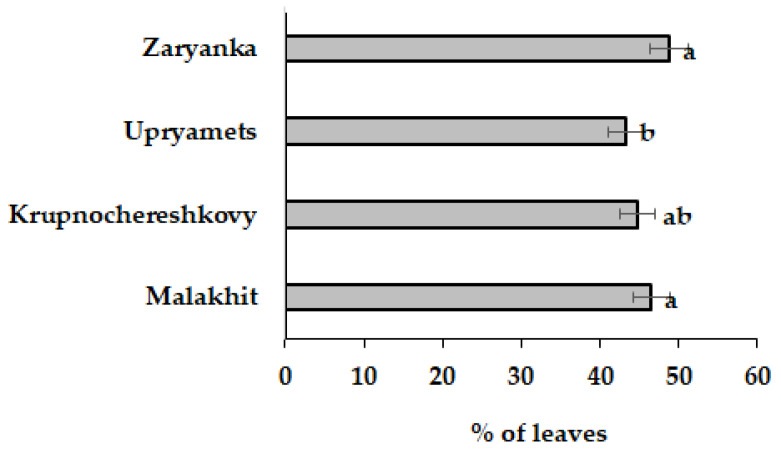
The leaves percentage in four rhubarb cultivars. Values with the same letters did not differ statistically according to Duncan test at *p* < 0.05.

Contrary to stalks, rhubarb leaves are usually discharged due to the high content of oxalic acid [13]. The stalk/leaves biomass ratio and the percentage of leaves biomass, out of the total biomass, represent important characteristics in further rhubarb processing. Indeed, the data presented in Table 1 indicate that the leaves/stalks biomass ratio increased from 1.05 (Zaryanka cv.) to 1.15 (Malakhit cv.), 1.23 (Krupnochereshkivy cv.) and 1.31 (Upryamets cv.). The percentage of unutilized leaves was in the range of 43.3–48.8%, with the lowest value in the Upryamets plants (Figure 1).

**Figure 2:**
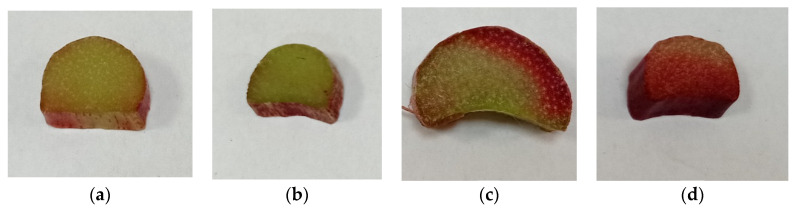
Appearance of rhubarb stalk slices of the four cultivars examined: (**a**) Malakhit, (**b**) Krupnochereshkovy, (**c**) Upryamets, (**d**) Zaryanka.

These results related to the significant morphological differences in the rhubarb stalks examined (Figure 2).

Furthermore, the cultivar Upryamets was also characterized by the highest stalk dry matter content, which is valuable in stalk pomace utilization.

### 3.2. Juice

#### 3.2.1. Juice Yield

To date, researchers have paid predominant attention to the production of rhubarb juice [1,32]. Indeed, depending on the production technology, its yield may reach up to 80–90%. In this respect, the utilization of precut rhubarb stalks is considered the utmost need to exclude rapid mill blocks due to their long stalk fibers [1]. The utilization of Robot coupe J 80 Ultra mill and stalks of approximately 4 cm in length in the present investigation resulted in a 72.6–82.4% juice yield (Table 2). Regarding this parameter, the data presented in Table 2 indicate the lack of statistically significant differences between the cultivars, with varietal differences not exceeding 4.7%.

#### 3.2.2. Nitrates

The cultivars examined showed low concentrations of juice nitrates, not exceeding 216 mg L^−1^ (Table 2), in contrast with the results of Will and Dietrich [1], who recorded nitrate levels as high as 816–893 mg·L^−1^. Genetic and environmental factors, such as water excess, nitrogen availability, light intensity and temperature, are known to greatly affect nitrate levels in plants [47,48]. In this respect, variations in the cultivars, habitat and climate characteristics in the present work and previous investigations [1] may explain the differences recorded. Furthermore, the comparison between rhubarb juice nitrate levels indicates the importance of this evaluation, as nitrates are known to be beneficial for heart care in moderate concentrations and risky to health at high content [47,48].

#### 3.2.3. Juice Density, Total Dissolved Solids (TDS) and Sugar Content

Among physicochemical characteristics of rhubarb juice, the density, total dissolved solids (TDS) and monosaccharides content demonstrated the lowest varietal differences (Table 2), which suggests that the values of these parameters are typical of rhubarb varieties grown in the same conditions. Similar low variations in sugar content were recorded by Mezeyova et al. [32] in five rhubarb cultivars. Among the cultivars tested, only cv. Malakhit showed a significantly lower total sugar level in the juice.

#### 3.2.4. Total Antioxidant Activity (AOA) and Total Polyphenol (TP) Content

Differently, genetic peculiarities highly affect rhubarb juice’s total antioxidant activity (AOA), concentration of polyphenols (TP) and anthocyanins (Table 2).

According to literature reports, red rhubarb stalks are the most popular in different countries due to their high levels of polyphenols and saccharides [2,28], which is consistent with the results of the present research. Indeed, among the four rhubarb cultivars examined, Zaryanka stalks were characterized by 3.24–4.76 times higher levels of anthocyanins and a 1.43–1.71 times higher total phenolics content compared to the other cultivars, and 1.58 times higher levels of total sugar than cv. Malakhit. The high variations in the anthocyanin content in the juice of the different rhubarb cultivars agreed with previous investigations [1,36,49], suggesting high prospects of the use of this quality indicator for rhubarb breeding.

On the other hand, it is worth mentioning the difficulties of comparing the present AOA and TP results with those recorded in literature due to different objects of investigation (stalks, juice) and methods of juice production [1], as well as the unknown growth management and genetic differences.

#### 3.2.5. Organic Acids

The most important characteristic of rhubarb juice is the total content of organic acids and their composition. Organic acids have a well-established role in plant growth, such as redox state modulation of cell compartments, storing phosphates and partaking in lignin biosynthesis, and are known to increase plant tolerance to environmental stress. Their content and composition are genetically governed; affect plant adaptability; provide antibacterial, antifungal and antiparasitic defense; and are directly related to juice quality [50,51]. The comparison between the results of our research with those of other researchers [1,13,32,52,53] has demonstrated the rather close values of the total organic acids content, within the range of 17–22 g·L^−1^. On the other hand, to date, the information about the organic acids composition of rhubarb juice/stalks is rather scant. In this respect, the investigations carried out in England in 1937 revealed the content of only three organic acids: oxalic, citric and malic [52]. In studies conducted in Romania in 2018, tartaric, oxalic, citric, malic and ascorbic acids were detected both in whole stalks [53] and juice [13]. Mezeyova et al. [32] found only malic acid in the juice of rhubarb grown in Slovenia (2021). Research performed in Germany in 2013 analyzed the juice levels of oxalic, citric and malic acids [1]. All these investigations reported a citric/malic ratio lower than one, with the lowest values recorded in Germany [1] and Slovenia [32] (0.12 and 0.13, respectively), and the highest in Romania [13,53] and England [52] (0.72).

Contrary to the mentioned data, the present results highlight the content of 10 organic acids in four cultivars of rhubarb, providing the first most complete organic acids profile of rhubarb juice. They indicate, for the first time, a significant amount of succinic acid in rhubarb juice and the interesting accumulation of sorbinic and benzoic acids, which are highly valuable compounds as preservatives in the food industry [54,55]. The highest level of succinic acid was detected in cv. Zaryanka juice, while the highest content of sorbinic and benzoic acids was detected in cv. Upryamets (Table 3).

Furthermore, the present results indicate that in the conditions of the Moscow region (Russia), the citric/malic acids ratio greatly exceeded one, reaching values of 156.5 (cv. Malakhit), 273.8 (cv. Krupnochereshkovy), 277.7 (cv. Zaryanka) and 452.5 (cv. Upryamets) (Table 3). The tendency for citric acid to be in excess (18.8) was recorded in our previous research on *Rheum tataricum* grown in the semi-desert area of Bogdinsko–Baskunchak Nature Reserve (Astrakhan region) [56]. The data in Table 3 indicate that rhubarb juice is characterized by the predominance of citric, oxalic and succinic acids with the total content accounting for 98% of the total organic acids amount, while the malic acid content did not exceed 0.2–0.5%. For juice production, high levels of organic acids improved ascorbic acid stabilization, whose concentration reached 125–164 mg L^−1^, indicating the high nutritional value of rhubarb juice.

**Figure 3:**
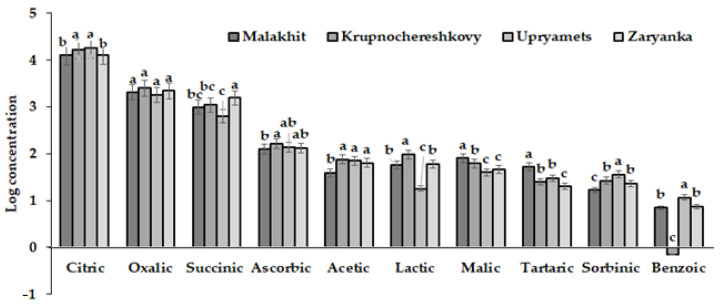
Juice organic acids profile of the four rhubarb cultivars examined. For each acid, values with the same letters did not differ statistically according to Duncan test at *p* < 0.05.

As can be observed in Table 3, the highest citric acid content was recorded in the cultivars Upryamets and Krupnochereshkovy, while Malakhit and Zaryanka displayed values that were lower by 1.5. Oxalic and ascorbic acid showed the most stable values, whereas the highest varietal differences occurred with lactic, tartaric and benzoic acids. In this respect, the highest levels of malic and tartaric acids, acetic and lactic acids, sorbic and benzoic acids and succinic acid were detected in the juice of the cultivars Malakhit, Krupnochereshkovy, Upryamets and Zaryanka, respectively. Overall, the cultivars Upryamets and Krupnochereshkovy proved to be the richest sources of organic acids (Figure 3).

Differences in the organic acid composition of rhubarb between the present study and previous investigations [1,13,32,52] may reflect varietal and environmental peculiarities. Slight differences in organic acid accumulation were recorded previously between different cultivars and plant densities [13,53]. The technological method of juice production may also influence the organic acid content in rhubarb juice [32]. In addition to the genetic factors, soil and environmental conditions such as temperature, mineral and water status may also affect organic acid synthesis [57]. The fruit citric acid accumulation is directly related to the respiration level, with high citrate levels at the early development stage [58,59,60]. The latter observation is in accordance with a previous investigation conducted by Allsopp [52], who found the highest levels of citric acid in rhubarb in April-June, characterized by intensive plant growth.

Nevertheless, future research is needed to further investigate the factors affecting the organic acid composition of rhubarb juice, among which the geographical habitat should be a priority considering the widespread distribution of rhubarb species in the world [2].

Moreover, it is worth highlighting the importance of assessing the organic acid composition of rhubarb juice, due to the great variations recorded in different areas in the world [1,13,32].

### 3.3. Stalk Pomace

Despite only 22.7% of stalk pomace is produced upon juice production, this residue has great importance (Table 4). Indeed, according to the biochemical analysis, the stalk pomace demonstrated high antioxidant activity, concentration of polyphenols and extremely high dietary fiber content.

According to Ooraikul et al. [61], the total content of dietary fiber in dry rhubarb pomace may reach 74%, of which the insoluble components account for 66%, while the soluble fibers only account for 8%. Investigations on laboratory animals revealed that rhubarb juice pomace decreased blood cholesterol and triglycerides levels under a cholesterol-rich diet [62]. This product (up to 5% out of the total food) did not cause changes in Ca accumulation [63]. The results of the present research indicate lower levels of the total dietary fiber (62.9%) compared to the literature data, which may relate to the differences in juice production technology. Differently, the soluble dietary fiber content was 1.8 times higher and reached 14.75%. Furthermore, the pectin content in the pomace samples was in the range of 21–24% which was significantly higher than that in the apple pomace (10–15%) and orange peel (20–30%), i.e., the main sources of industrial pectin production [64]. Thanks to its gelling properties, pectin is widely used in the food industry and in pharmaceutical branches as a carrier of drugs released in the intestinal tract. Furthermore, the nonsignificant species differences in the dietary fiber content, and particularly pectin, open new prospects of industrial rhubarb pomace utilization, with a neglectable cultivar role.

Overall, both rhubarb pomace and juice should be considered powerful functional food products.

### 3.4. Rhubarb Roots

Rhubarb roots are highly valuable in traditional medicine due to their anti-carcinogenic, anti-inflammatory and antidiabetic effects [2]. According to our results, they have the highest content of antioxidants, which is consistent with previous investigation on *R. tataricum* [56]. The data presented in Table 2, Table 4 and Table 5 indicate, that the AOA activity gradually decreased from the root pulp to the root peel, juice pomace and juice, while the rhubarb roots showed the highest nitrate content. Nevertheless, the latter fact does not cause health risks due to the low doses used in medicine, and it may be considered a beneficial factor for heart care [65]. Among the cultivars tested, Upryamets and Zaryanka were characterized by the highest levels of root total antioxidant activity (AOA), suggesting the significance of varietal differences for pharmaceutical purposes. Furthermore, significant differences in the antioxidant status of the root peel and pulp, recorded in our research for the first time, may become valuable to produce drugs with high antioxidant defense. Further studies are needed to unveil possible applications of the revealed phenomenon.

## 4. Conclusions

The results of the present investigation prove the importance of the complex rhubarb processing to produce functional food and additives with beneficial medicinal effects, characterized by the high value of the juice’s organic acid composition, the pomace’s dietary fiber and pectin and root antioxidants. However, this processing is influenced by the varietal differences in the total antioxidant activity (AOA); organic acid composition and content; leaf, stalk and root biomass; and anthocyanin accumulation levels.

## Figures and Tables

**Table 1:** Yield and biometric parameters of four rhubarb cultivars.

Parameter	Malakhit	Krupnochereshkovy	Upryamets	Zaryanka	M ± SD	CV (%)
Leaves biomass						
(g per plant)	822.3 ± 70.2 ^b^	771.2 ± 63.2 ^bc^	1736.5 ± 109.0 ^a^	689.4 ± 55.3 ^c^	1005 ± 491	48.9
Stalk biomass						
(g per plant)	946.5 ± 78.0 ^b^	951.8 ± 79.8 ^b^	2277.3 ± 200.0 ^a^	722.3 ± 67.2 ^c^	1224 ± 710	58.0
Stalk yield (t·ha^−1^)	56.79 ± 5.32 ^b^	57.11 ± 5.65 ^b^	79.64 ± 7.89 ^a^	43.34 ± 4.29 ^c^	59.2 ± 15.0	25.3
Stalk width (cm)	1.8 ± 0.1 ^b^	1.9 ± 0.2 ^b^	4.0 ± 0.3 ^a^	1.7 ± 0.2 ^b^	2.4 ± 1.1	46.8
Stalk length (cm)	59.0 ± 3.6 ^a^	57.1 ± 3.3 ^a^	61.0 ± 4.6 ^a^	56.1 ± 3.4 ^a^	58.0 ± 2.0	3.4
Stalk dry matter (%)	6.4 ± 0.5 ^b^	7.5 ± 0.6 ^ab^	8.5 ± 0.7 ^a^	7.3 ± 0.6 ^ab^	7.4 ± 0.9	12.2

Along each line, values with the same letters did not differ statistically according to Duncan test at *p* < 0.05.

**Table 2:** Varietal differences in biochemical characteristics of rhubarb juice.

Juice Parameter	Malakhit	Krupnochereshkovy	Upryamets	Zaryanka	M ± SD	CV (%)
Yield (%)	74.6 ± 7.5 ^a^	74.9 ± 7.5 ^a^	82.4 ± 7.9 ^a^	77.2 ± 7.6 ^a^	77.3 ± 3.6	4.7
Density (g·mL^−1^)	1.114 ± 0.001 ^a^	1.115 ± 0.001 ^a^	1.115 ± 0.001 ^a^	1.109 ± 0.001 ^a^	1.113 ± 0.003	0.3
Brix°	0.7 ± 0.1 ^c^	1.8 ± 0.1 ^a^	1.6 ± 0.1 ^b^	1.7 ± 0.1 ^c^	1.5 ± 0.2	13.3
Nitrates (mg·L^−1^)	175 ± 16 ^b^	190 ± 18 ^ab^	216 ± 20 ^a^	174 ± 17 ^b^	189 ± 20	10.6
TDS (mg·L^−1^)	397 ± 35 ^a^	396 ± 35 ^a^	426 ± 40 ^a^	386 ± 36 ^a^	401 ± 17	4.2
Monosaccharides (%)	0.96 ± 0.10 ^a^	1.07 ± 0.10 ^a^	1.02 ± 0.10 ^a^	1.10 ± 0.10 ^a^	1.04 ± 0.06	5.8
Total sugar (%)	1.28 ± 0.11 ^b^	2.10 ± 0.20 ^a^	1.95 ± 0.17 ^a^	2.00 ± 0.20 ^a^	1.83 ± 0.37	20.2
AOA (mg GAE ·mL^−1^)	4.4± 0.4 ^b^	4.8 ± 0.5 ^b^	4.8 ± 0.5 ^b^	7.6 ± 0.7 ^a^	5.4 ± 1.5	27.8
TP (mg GAE · mL^−1^)	3.1 ± 0.3 ^b^	3.7 ± 0.3 ^b^	3.3 ± 0.3 ^b^	5.3 ± 0.5 ^a^	3.9 ± 1.0	25.6
Anthocyanins (mg·L^−1^)	2.5 ± 0.2 ^b^	1.9 ± 0.2 ^c^	1.7 ± 0.1 ^c^	8.1 ± 0.8 ^a^	3.6 ± 3.1	86.1

AOA: total antioxidant activity; TP: total polyphenol content; TDS: total dissolved solids. Along each line, values with the same letters did not differ statistically according to Duncan test at *p* < 0.05.

**Table 3:** Varietal differences in organic acid content in rhubarb juice (mg L^−1^).

Organic Acid	Malakhit	Krupnochereshkovy	Upryamets	Zaryanka	M ± SD	CV (%)
Citric	12550 ± 890 ^b^	17000 ± 1300 ^a^	18100 ± 1650 ^a^	12800 ± 1008 ^b^	15113 ± 2852	18.9
Oxalic	2045 ± 175 ^a^	2520 ± 223 ^a^	1800 ± 175 ^a^	2200 ± 201 ^a^	2141 ± 301	14.1
Succinic	993 ± 88 ^bc^	1105 ± 108 ^ab^	635 ± 63 ^c^	1550 ± 138 ^a^	1071 ± 377	35.2
Ascorbic	125.0 ± 12.0 ^b^	164. ± 16.1 ^a^	139.0 ± 13.0 ^ab^	133.0 ± 12.3 ^ab^	140.3 ± 16.8	12.0
Acetic	39.1 ± 4.0 ^b^	75.0 ± 7.2 ^a^	70.1 ± 6.9 ^a^	64.0 ± 6.1 ^a^	62.0 ± 16.0	25.8
Lactic	58.0 ± 5.1 ^b^	95.0 ± 9.2 ^a^	18.1 ± 1.5 ^c^	60.2 ± 6.0 ^b^	57.8 ± 31.5	54.5
Malic	80.2 ± 8.0 ^a^	62.1 ± 6.1 ^b^	40.0 ± 3.9 ^c^	46.1 ± 5.0 ^c^	57.0 ± 17.9	31.4
Tartaric	52.1 ± 5.0 ^a^	25.0 ± 2.2 ^b^	30.1 ± 3.0 ^b^	20.0 ± 2.0 ^c^	31.8 ± 14.1	44.3
Sorbinic	16.8 ± 1.3 ^c^	26.7 ± 2.6 ^b^	36.2 ± 3.5 ^a^	23.1 ± 2 ^b^	25.7 ± 8.1	31.5
Benzoic	7.0 ± 0.6 ^b^	0.7 ± 0.1 ^c^	11.7 ± 1.1 ^a^	7.4 ± 0.7 ^b^	6.7 ± 4.5	67.2
Total	15966.2 ^c^	21073.5 ^a^	20880.2 ^ab^	16903.8 ^bc^	18706 ± 3384	18.1

Along each line, values with the same letters did not differ statistically according to Duncan test at *p* < 0.05.

**Table 4:** Biochemical characteristics of stalk pomace.

Parameter	Malakhit	Krupnochereshkovy	Upryamets	Zaryanka	M ± SD	CV (%)
AOA (mg GAE ·g^−1^ d.w.)	31.5 ± 3.0 ^a^	30.5 ± 3.0 ^a^	28.3 ± 2.9 ^a^	34.4 ± 3.2 ^a^	31.2 ± 2.5	8.0
TP (mg GAE ·g^−1^ d.w.)	18.8 ± 1.8 ^a^	15.8 ± 1.5 ^b^	17.0 ± 1.7 ^ab^	18.1 ± 1.8 ^ab^	17.4 ± 1.3	7.5
TDS (g·kg^−1^ d.w.)	46.1 ± 4.5 ^a^	50.0 ± 4.9 ^a^	37.4 ± 3.7 ^b^	43.2 ± 4.2 ^ab^	44.0 ± 5.0	11.4
Nitrates (mg·kg^−1^ d.w.)	2045 ± 202 ^ab^	2123 ± 200 ^a^	1335 ± 129 ^c^	1682 ± 164 ^b^	1796 ± 363	20.2
Nonsoluble Fiber (% d.w.)	47.8 ± 4.8 ^a^	44.9 ± 4.5 ^a^	51.9 ± 5.2 ^a^	47.8 ± 4.8 ^a^	48.1 ± 2.9	6.0
Soluble Fiber (% d.w.)	15.8 ± 1.6 ^a^	13.9 ± 1.4 ^a^	14.9 ± 1.5 ^a^	14.4 ± 1.4 ^a^	14.9 ± 0.8	5.5
Total Fiber (% d.w.)	63.6 ± 6.1 ^a^	58.8 ± 5.8 ^a^	66.8 ± 6.6 ^a^	62.2 ± 6.0 ^a^	62.9 ± 3.3	5.3
Pectin (% d.w.)	23.0 ± 2.1 ^a^	21.0 ± 2.0 ^a^	24.0 ± 2.3 ^a^	23.0 ± 2.0 ^a^	22.8 ± 1.3	5.5

AOA: total antioxidant activity; TP: total polyphenol content; TDS: total dissolved solids. Along each line, values with the same letters did not differ statistically according to Duncan test at *p* < 0.05.

**Table 5:** Antioxidant status and nitrate content in rhubarb roots.

Parameter	Malakhit	Krupnochereshkovy	Upryamets	Zaryanka	M ± SD	CV (%)
AOA peel (mg GAE ·g^−1^ d.w.)	121.1 ± 12.0 ^d^	115.0 ± 11.0 ^d^	170.1 ± 16.2 ^bc^	150.0 ± 15.1 ^c^	139.0 ± 26.1	18.7
AOA pulp (mg GAE ·g^−1^ d.w.)	164.0 ± 16.1 ^c^	161.2 ± 15.0 ^c^	192.1 ± 18.0 ^b^	232.0 ± 20.1 ^a^	187.3 ± 33.0	17.6
TP peel (mg GAE ·g^−1^ d.w.)	16.6 ± 1.6 ^b^	17.8 ± 1.7 ^b^	19.8 ± 1.9 ^b^	19.5 ± 1.8 ^b^	18.4 ± 1.5	8.2
TP pulp (mg GAE ·g^−1^ d.w.)	20.0 ± 1.9 ^ab^	20.6 ± 2.0 ^ab^	21.0 ± 2.0 ^ab^	24.9 ± 2.3 ^a^	21.6 ± 2.2	10.2
Nitrates (mg kg^−1^ d.w.)	664 ± 66 ^a^	558 ± 54 ^ab^	465 ± 46 ^b^	474 ± 46 ^b^	540 ± 93	17.2

AOA: total antioxidant activity; TP: total polyphenol content. Along each line, values with the same letters did not differ statistically according to Duncan test at *p* < 0.05.

## Data Availability

Not applicable.
